# Laryngocele: a cause of upper airway obstruction

**DOI:** 10.1016/S1808-8694(15)30765-5

**Published:** 2015-10-19

**Authors:** Jose Antonio de Paula Felix, Felippe Felix, Luiz Fernando Pires de Mello

**Affiliations:** 1 Adjunct Professor of Otorhinolaryngology - Federal University of Rio de Janeiro; 2 2nd year Resident in Otorhinolaryngology - Clementino Fraga Filho University Hospital - Federal University of Rio de Janeiro; 3 MD. Head of the Head and Neck Surgery Service - Bonsucesso General Hospital

**Keywords:** laryngocele, cervical swelling, upper airway obstruction

## Abstract

Laryngoceles are abnormal dilatations of the laryngeal saccule, which rises between the ventricular folds, the base of the epiglottis and the inner surface of the thyroid cartilage. Clinical symptoms are rare, and the find of asymptomatic laryngoceles in pathology studies are frequent. Sometimes it is presented as cervical swelling causing airway obstruction in need of emergency intervention. In this study, we report a case of upper airway obstruction due to laryngocele treated by emergency tracheotomy and we review of the literature. Laryngocele complications include infection (pyocele formation), pathogens aspirations with subsequent bronchitis and pneumonia and upper airway obstruction, as in the case reported. Despite being benign tumors, laryngoceles cause relevant airway obstruction. Correct diagnosis and proper treatment can preclude emergencies as it happened to our patient hereby reported.

## INTRODUCTION

The saccule or laryngeal ventricle appendage is usually present in most human larynxes. It exits through the anterior border of the ventricle and extends superiorly through the Paralaryngeal space, having the ventricular fold medially and the thyroid cartilage laterally. Laryngoceles are dilations of the Morgagni's ventricle sacculus, filled with air that communicate with the laryngeal lumen and may be temporarily distended by mucus.

Clinical relevance is rare and it is common to find laryngoceles in postmortem exams of asymptomatic people. In other cases it may present itself as a large cervical mass that blocks the airways and needs urgent care. In this study, the authors report a case of laryngocele causing airway obstruction, requiring urgent tracheostomy, and they review the literature on this topic.

## CASE REPORT

**Figure 1:**
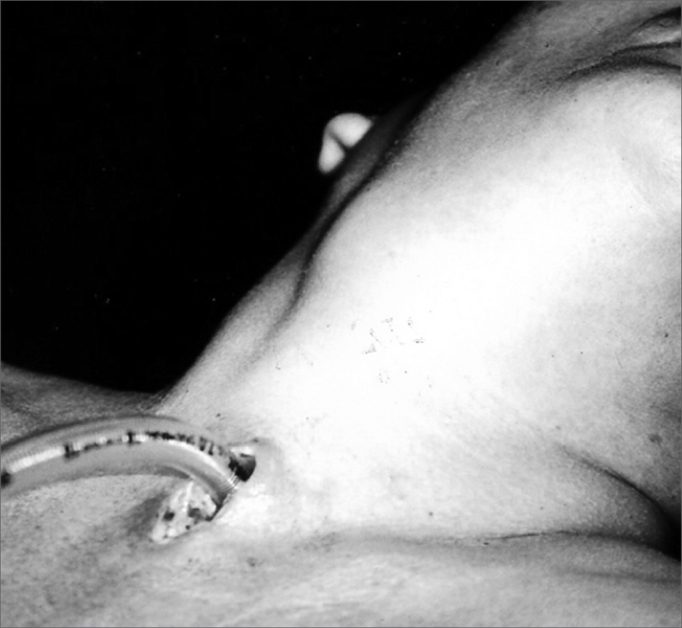
Laryngocele's external part. Notice the neck mass on the right. At this point the patient is already tracheostomized.

**Figure 2:**
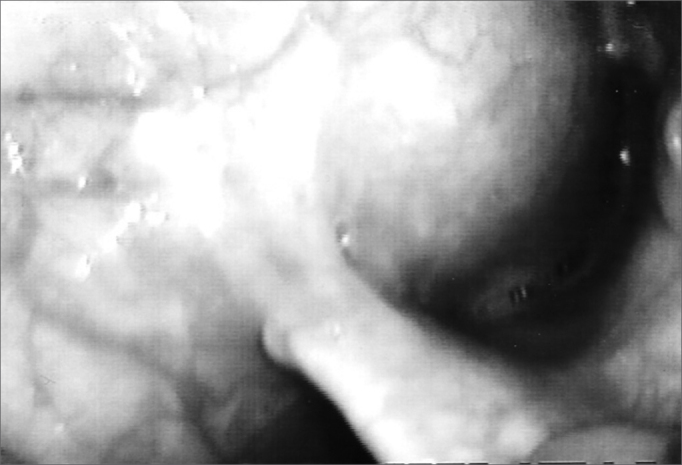
Laryngocele's internal part. Videolaryngoscopy showing the tumor occluding the laryngeal vestibule, hiding the piriform sinus.

**Figure 3:**
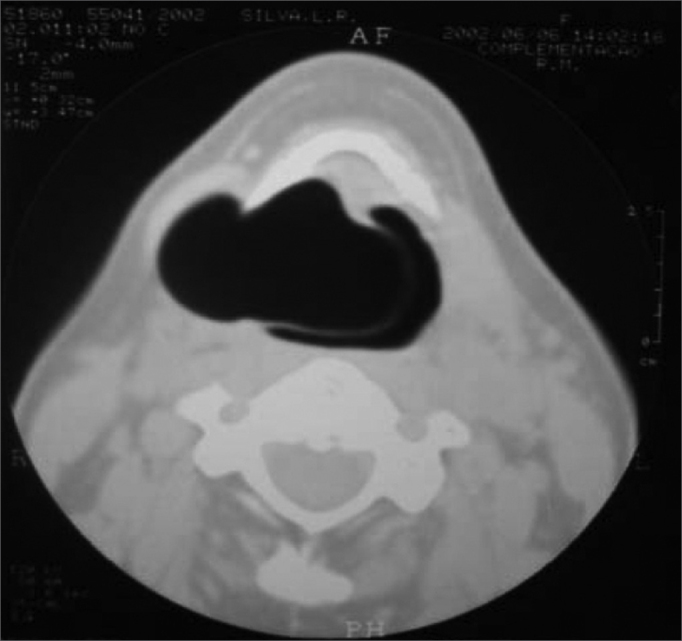
Larynx CT scan showing the laryngocele.

**Figure 4:**
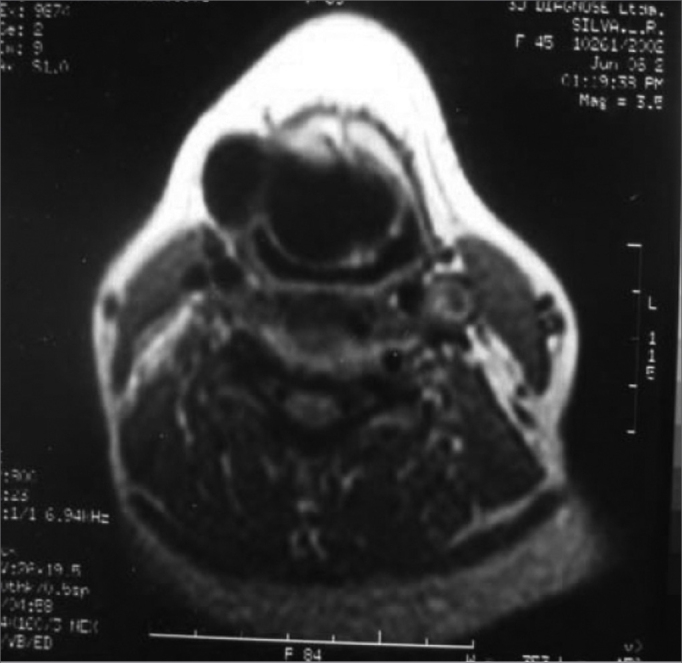
MRI showing the laryngocele in details.

**Figure 5:**
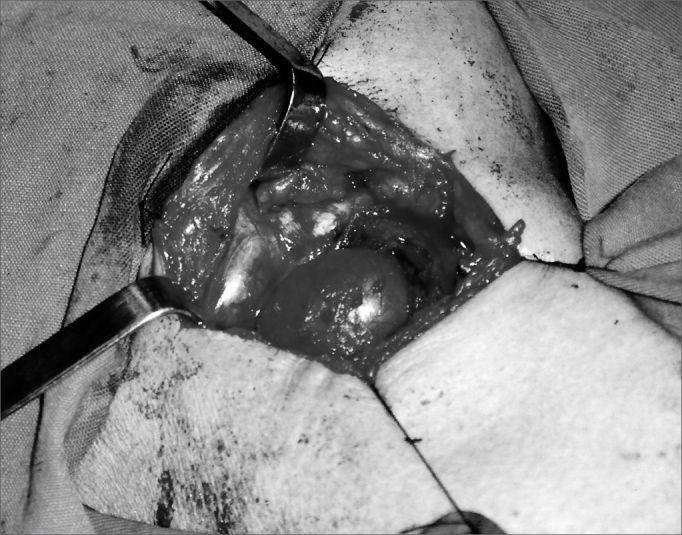
Lesion removal by an external approach.

**Figure 6:**
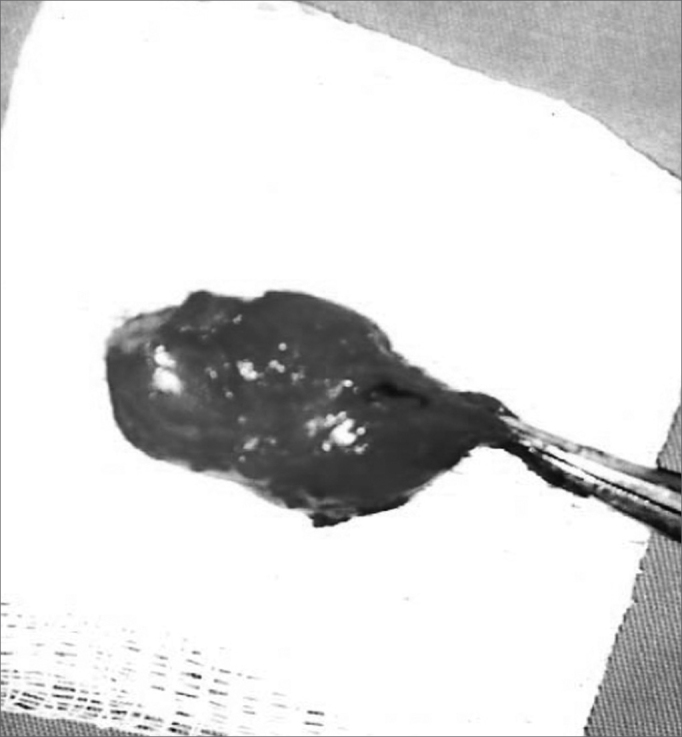
Surgical specimen.

**Figure 7:**
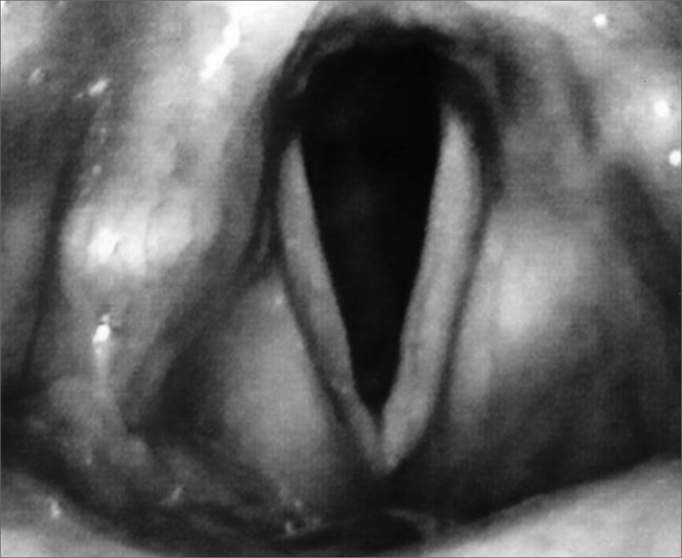
Postoperative Video-laryngoscopy.

Our patient was a 45 year old female, housewife, seen in May of 2002, complaining of dysphonia for 40 days, associated with snoring. She did not smoke or had any pulmonary disorder, nor had she had prior laryngeal problems. During physical exam, she had a mass in her right cervical region (Figure 1) and during the laryngoscopic exam she had a bulging near the ventricular fold and the right aryepiglottic fold (Figure 2). We ordered a CT scan and an MRI (Figure 3, Figure 4) which revealed a large cystic lesion filled with air, thus confirming the diagnosis of laryngocele. In late June of 2002, while she underwent preoperative exams, she was having intense dyspnea and required urgent tracheotomy. As her clinical signs stabilized, she underwent resection of the lesion by an external via (Figure 5, Figure 6). She progressed without complications in her postoperative, with full symptoms improvement and normal laryngoscopic exam (Figure 7).

## DISCUSSION

Virchow, in 1867, was the first to call this abnormal dilation of the laryngeal ventricle a laryngocele, however the first report of such disorder came in 1829, from a French military surgeon - Dominique Larrey.

Laryngoceles may expand medially, causing a reduction in the supraglottic space called internal laryngocele. Other times it may expand laterally, reducing the supraglottic space, thus being called internal laryngocele. Other times it may expand laterally, exiting through the thyroid membrane near the internal branch of the upper laryngeal nerve, causing neck bulging, thus being called external laryngocele. About half of the laryngoceles are of the mixed type, having both the internal and the external components. It is usually unilateral, being bilateral in only 15%.

Laryngoceles are more commonly found in men in a 5:1 ratio, in their fifth or sixth decades of life. Its cause is unknown, however it is associated with chronic cough, blowing in musical instruments, glass blowing and laryngeal carcinoma. Histologically we find a ciliated pseudostratified cylindrical epithelium with a varied number of goblet cells on a thin basal membrane^1^, ^2^, ^3^.

There is much controversy regarding the etiology of laryngoceles4. Its origins involve congenital factors, such as laryngoceles in neonates; and also acquired factors. In adults, a congenital defect or an anatomical variation of the sacculus may be the cause, as are acquired factors such are the cases of pharyngeal or laryngeal carcinomas, and people whom occupation or leisure involve raising intralaryngeal pressure, such as blowing musical instruments. However, some factors do not corroborate this statement, such as the fact that many patients do not have any predisposing factor and most of these alterations are unilateral. Laryngeal carcinoma is very likely associated with an increase in intraluminal pressure, both by obstruction of upper airways, speech effort, excessive cough and local mechanical conditions. Thus, it is important to carry out an investigation in patients with laryngocele aiming at ruling out any associated malignancy. In 1995, Thomé et al., described nine cases of laryngoceles and saccular cysts in the following patients: an army horn player, two smokers with chronic cough and two patients who used their ventricular folds to speak.

Symptoms may be divided according to the type of laryngocele. In the internal laryngoceles they may interfere in speech production and cause snoring or hoarseness, and even upper airway obstruction as the case hereby presented. Other symptoms are: a foreign body sensation, sore throat and cough. In cases of external laryngoceles of the mixed type, there will be a neck mass with or without laryngeal symptoms associated.

A CT scan can help distinguish between cysts filled with air from those filled with liquid and may detect a mixed laryngocele in which only one of the components, internal or external, was clinically suspected.

Differential diagnosis include: saccular cyst, branchial cyst, neck abscess and lympho-adenopathy. Saccular cysts do not communicate with the laryngeal lumen, and it is usually filled with fluid^6^^,^^7^^,^^8^.

Laryngocele complications include infection (pyocele), pathogens aspiration and subsequent bronchitis and pneumonia, infection in the lateral larynx (after rupture) and upper airway obstruction, as is the case hereby presented. In 1997, Pinto et al., described a similar case that also caused upper airway obstruction, however the patient also had chronic obstructive pulmonary disease, and this made things even worse as far as respiratory failure is concerned^9^.

Laryngocele treatment will depend on disease size and repercussion. Small internal laryngoceles and saccular cysts may be removed endoscopically or transendoscopically excised with laser, causing less edema and less post-operative adherence when compared with the conventional method. Small and recurrent internal laryngoceles, which may be associated with malignancy and large internal or external laryngoceles are removed by an external approach. Small, asymptomatic laryngoceles are followed up and only removed in case they become symptomatic or cause some cosmetic alteration^10^^,^^11^.

## FINAL COMMENTS

Despite representing benign disorders, laryngoceles are a potential cause for respiratory obstruction that may threaten the patient's life. Proper diagnosis and handling may avoid an emergency situation, as was the case hereby presented.
